# Management and Sequelae of Intruded Anterior Primary Teeth: A Systematic Review

**DOI:** 10.5005/jp-journals-10005-1371

**Published:** 2016-09-27

**Authors:** Deepa Gurunathan, Muthu Murugan, Sujatha Somasundaram

**Affiliations:** 1 Reader, Department of Pedodontics, Saveetha Dental College and Hospital, Chennai, Tamil Nadu, India; 2 Professor, Department of Pedodontics, Sri Ramachandra Dental College and Hospital, Chennai, Tamil Nadu, India; 3 Reader, Department of Pedodontics, Saveetha Dental College and Hospital, Chennai, Tamil Nadu, India

**Keywords:** Management, Primary anterior teeth intrusion, Sequelae.

## Abstract

**Objective:**

The objective of this systematic review is to analyze the various treatment modalities and sequelae of intruded anterior primary teeth.

**Materials and methods:**

Electronic search in PubMed, Cochrane, and Science Direct databases was done. Hand search was performed using the reference list of chosen articles from electronic search. Three reviewers analyzed the articles independently, assessed the quality of the studies, and derived data.

**Results:**

Ten case series were identified from the electronic and hand search. No randomized control studies were available. In the observational studies treatment of intruded primary teeth ranged from conservative management, which includes waiting for spontaneous re-eruption as well as repositioning to invasive procedure, such as extraction.

**Conclusion:**

Spontaneous eruption is a treatment option of intruded primary teeth in absence of damage to a permanent tooth. Surgical repositioning of intruded primary teeth has also shown as a viable alternative treatment modality. Extraction to be performed if complications develop.

**How to cite this article:**

Gurunathan D, Murugan M, Somasundaram S. Management and Sequelae of Intruded Anterior Primary Teeth: A Systematic Review. Int J Clin Pediatr Dent 2016;9(3):240-250.

## INTRODUCTION

During the initial growing period of a child when motor coordination is not well developed, the incidence of trauma to the primary dentition is greatest.^1^ The highly resilient and flexible supporting structures result in luxation injuries rather than fractures of the teeth.^2^ These luxation injuries constitutes 62 to 73% of all injuries to the primary dentition of which intrusive and extrusive traumas are more common than other luxation injuries.^3-14^

Intrusive injuries account for 4 to 22% of damage to the primary teeth.^3,5,11,14-21^ Intrusion is defined as an apical displacement of tooth into the alveolar bone along with compression of the periodontal ligament with or without fracture of the alveolar socket.^22^

Coronal discoloration, obliteration of pulp canal, pulpal necrosis, internal resorption, pathological root resorption or lack of re-eruption due to ankylosis, and spontaneous re-eruption back to its original position or ectopically are the sequelae of an intruded primary tooth.^14-21^ Due to anatomic proximity of the developing permanent tooth germ to the primary root apex, an intruded primary tooth can cause developmental disturbances to the successor tooth.^23,24^

Factors, such as child’s maturity and ability to cope up with the emergency situation, presence of oral habits, and the time of exfoliation of injured tooth determine the treatment plan.^25^ The degree of intrusion, relation between the root apex of primary tooth and the permanent tooth, degree of development of the root of intruded tooth, signs of previous injury, number of teeth intruded, associated injuries, condition of surrounding alveolar bone, presence of caries in the affected teeth, and time elapsed since the injury also influence the treatment plan. A complete medical and dental history of trauma is essential for an accurate diagnosis of the injured tooth. In addition, treatment of an intruded primary tooth depends on thorough clinical and radiographic examination.^26^

Intruded primary teeth are being managed in a wide array of modalities ranging from observation for spontaneous re-eruption, surgical repositioning, or extrac-tion.^3,11,14,16-21,25^ Though various guidelines are available for the treatment of intruded primary incisors, optimal treatment for intruded primary teeth has always been a topic of controversy among clinicians.^22,26^ Treatment of intrusion is usually limited to extraction due to danger underlying the permanent tooth bud or young age. The objective of this systematic review of literature is to analyze systematically the various treatment modalities and sequelae of intruded anterior primary teeth.

**Table 1:** **Table 1:** Search terms used in PubMed

*Sl. no.*		*Search terms*	
1		(“therapeutics” [MeSH Terms] OR “therapeutics” OR “therapeutic”) AND approach AND intruded AND (“tooth, deciduous” [MeSH Terms] OR (“tooth” AND “deciduous”) OR “deciduous tooth” OR (“primary” AND “tooth”) OR “primary tooth”) “therapy” OR “therapy” OR “treatment” OR “therapeutics”	
2		(“organization and administration” [MeSH Terms] OR (“organization” AND “administration”) OR “organization and administration” OR “management” OR “disease management” [MeSH Terms] OR (“disease” AND “management”) OR (“disease management”) AND intruded AND primary AND (“incisor” [MeSH Terms] OR “incisor” OR “incisors”)	
3		extraction AND intruded AND (“tooth, deciduous” [MeSH Terms] OR (“tooth” AND “deciduous”) OR “deciduous tooth” OR (“primary” AND “tooth”) OR (“primary tooth”)	
4		repositioning AND intruded AND (“tooth, deciduous” [MeSH Terms] OR (“tooth” AND “deciduous”) OR “deciduous tooth” OR (“primary” AND “tooth”) OR “primary tooth”)	
5		pulpal AND obliteration AND intruded AND (“tooth, deciduous” [MeSH Terms] OR (“tooth” AND “deciduous”) OR “deciduous tooth” OR (“primary” AND “tooth”) OR “primary tooth”)	
6		(“dental pulp necrosis” [MeSH Terms] OR (“dental” AND “pulp” AND “necrosis”) OR “dental pulp necrosis” OR (“pulpal” AND “necrosis”) OR “pulpal necrosis”) AND intruded AND (“tooth, deciduous” [MeSH Terms] OR (“tooth” AND “deciduous”) OR “deciduous tooth” OR (“primary” AND “tooth”) OR “primary tooth”)	
7		(“ankylosis” [MeSH Terms] OR “ankylosis”) AND intruded AND (“tooth, deciduous” [MeSH Terms] OR (“tooth” AND “deciduous”) OR “deciduous tooth” OR (“primary” AND “tooth”) OR “primary tooth”)	
8		(“diagnosis” [Subheading] OR “diagnosis” OR “diagnosis” [MeSH Terms]) AND intruded AND (“tooth, deciduous” [MeSH Terms] OR (“tooth” AND “deciduous”) OR “deciduous tooth” OR (“primary” AND “tooth”) OR “primary tooth”)	

## MATERIALS AND METHODS

A comprehensive dental literature search was made electronically from PubMed, Cochrane Database of Systematic Review, and Science Direct up to June 2014. Later, hand searching of reference list of articles that were obtained from electronic search that compiled with inclusion and exclusion criteria was also included. Table 1 shows the key words and their combinations used in the literature search. Explicit inclusion and exclusion criteria were used to identify the relevant literature to be included in the review. Study selection was done at title, abstract, and full text screening stage by employing the inclusion and exclusion criteria. Three reviewers did the search independently and arrived at a consensus after discussion regarding the selection of articles (Flow Chart 1).

### Inclusion Criteria

- Case series and cohort studies on various management techniques of intrusion of primary anterior teeth.
- Studies that evaluated the sequelae of intruded primary tooth.

### Exclusion Criteria

- Articles that did not meet the inclusion criteria, and unpublished data were excluded.
- Literature in languages other than English was not considered in the review.
- We also excluded letters to editors, editorials, review articles, and commentaries; however, we did read them to identify any potential information relevant to our review.
- Articles that evaluated postextraction complications of intruded primary teeth.

**Flow Chart 1:**
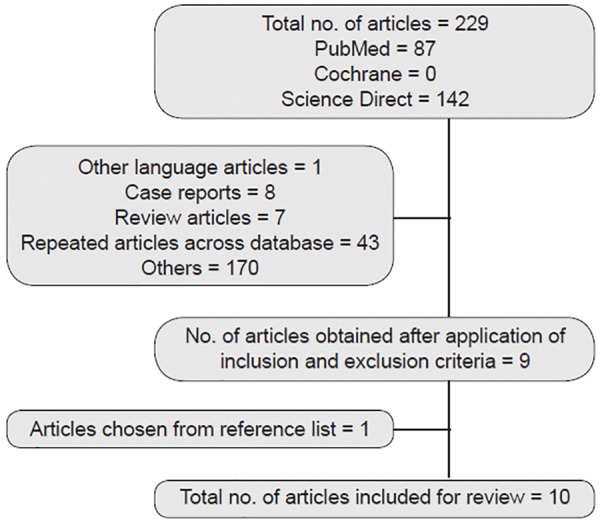
Selection of articles

### Data Extraction

Data extraction was done on a specially designed data extraction form by the three authors independently. Data collection was undertaken according to the following criteria.

*Participants:* Country; age; radiograph taken - Yes/No; labial bone fracture - Yes/No

*Intervention:* Antibiotic therapy; observation with oral hygiene instructions; extraction; surgical repositioning *Outcome:* Re-eruption of tooth; pulpal status; and peri-odontal status of the intruded primary tooth.

*Study design:* Case series; observational study - both prospective and retrospective studies.

### Data Synthesis

Information regarding the various treatment procedures according to the level of intrusion of primary anterior teeth and their sequela was extracted. Data regarding level of spontaneous re-eruption either complete, partial, or no re-eruption was recorded. In addition, status of teeth that were surgically repositioned, and teeth that were extracted immediately or due to late complications was recorded. Pulpal and periodontal complications of intruded primary teeth were also synthesized. They were:

- Pulpal status which included pulpal necrosis and obliteration of the canal
- *Periodontal status:* Periodontal breakdown; mobility; ankylosis
- Coronal discoloration either dark or yellow
- *Resorption:* Internal or external resorption of the intruded primary tooth.

### Study Analysis

Quality Assessment and control of bias was done by three authors using the methodological checklist for prognostic studies developed by National Institute for Health and Care Excellence of United Kingdom^27^ (Table 2). Each study was evaluated for all the criteria and was classified to have either “yes,” “no,” or “unclear” methodology to minimize risk of bias. If a study fulfills at least five criteria as “yes,” the study is said to have high methodological quality; if the study fulfils at least three criteria as “yes,” the study is said to have moderate methodological quality. Study that fulfils two or less criteria with “yes” was classified to have low methodological quality.

## RESULTS

The electronic search of PubMed, Cochrane, and Science Direct yielded 105 citations. Following deletion of repeated articles and those that did not meet inclusion criteria, 9 relevant papers were obtained. Additionally, 1 more paper was procured on screening the references of the reviews and selected articles. Therefore, a total of 10 observational studies^3,11,14,16-21,25^ were identified for this systematic review. All the studies were Level 4 according to levels of evidence.^28^

Different authors have followed different systems to classify intrusion injuries which are enlisted below.

### Carvalho et al^16^

*Total intrusion:* Tooth is completely inside the gingival tissue. *Partial intrusion:* Part of the tooth is inside the gingival.

### Colak et al^20^

*Mild:* Less than 2 mm

*Moderate:* 2 to 4 mm

*Severe:* Greater than 4 mm.

### Altun et al^21^

Shortening of the crown by at least one-half of its clinical size is considered as intruded.

### Radiographs as a Diagnostic Tool to assess the Intruded Anterior Primary Teeth

In all the studies, intraoral periapical radiographs were taken to assess the position of intruded anterior primary teeth immediately following trauma.^3,11,14-16,21,25^ Only in cases of very young and uncooperative children, radiographs were not taken.

### Association of Treatment and Sequelae of Intruded Anterior Primary Teeth

Three authors reviewed the sequelae of intruded primary teeth (Table 3). Ravn^3^ found that majority of the intruded teeth re-erupted spontaneously (80/88 teeth). Pulp canal obliteration was seen in 30 teeth and periodontal breakdown in 10 teeth as late complications. Soporowski et al^11^ found that treatment rendered was significantly associated with the sequelae of intruded teeth. Even though Soporowski et al^11^ has compared spontaneous re-erupted teeth and repositioned teeth, definite conclusions cannot be drawn as number of teeth repositioned were only 3 when compared to 21 teeth that were allowed for spontaneous re-eruption.^11^ Hirata et al^25^ observed favorable prognosis with no pathological pulpal changes, and mobility in teeth allowed for spontaneous re-eruption.

### Spontaneous Re-eruption

Degree of intrusion and spontaneous re-eruption (Table 4):

In four of ten studies, it was seen that degree of intruded teeth did not have any effect on re-eruption.^3,16,17,25^ Ravn^3^ and Colak et al^20^ observed that completely or severely intruded teeth re-erupted within 4 to 6 months, whereas partially or mild-to-moderate intruded teeth re-erupted within 2 to 4 months. Holan and Ram^17^ found no significant difference with degree of intrusion.

Age of injury as a factor in spontaneous re-eruption: Only three of the nine studies that were included for the review gave an association between the age at the time of injury and spontaneous re-eruption.^17,20,21^ These three studies have shown that the possibility of spontaneous re-eruption is greater in children injured around 2 years and younger. Holan and Ram^17^ found that teeth re-erupt in ectopic position when children were injured at 24 to 35 months.

**Table 2:** **Table 2:** Analysis of studies

		*Ravn^3^*		*Soporowski* * etal^11^*		*Borum and* * Andreasen^14^*		*Carvalho* * etal^16^*		*Holan and* * Ram^17^*		*Gondim and* * Moreira Veto^18^*		*Spinas* * et al^19^*		*Colak et al^20^*		*Altun et al^21^*		*Hirata et al^25^*	
The study sample represents the population of interest		Unclear		Unclear		Unclear		Unclear		Unclear		Unclear		Unclear		Unclear		Unclear		Unclear	
Loss to follow up is unrelated to key characteristics		No		No		Yes		Yes		No		Yes		No		Yes		Yes		Yes	
The prognostic factor of interest is adequately measured		Unclear		Yes		No		Yes		Yes		Yes		No		Yes		Unclear		Yes	
The outcome of interest is adequately measured		Yes		Unclear		Yes		Yes		Yes		Yes		No		Yes		Yes		Yes	
Important potential confounders are appropriately accounted for		No		No		No		No		Yes		No		No		No		No		Yes	
The statistical analysis is appropriate for the design of the study		Unclear		Unclear		Unclear		Unclear		Yes		Unclear		Unclear		No		Yes		Yes	
Category and situation of the article		Low methodology		Low methodology		Low methodology		Moderate methodology		Moderate methodology		Moderate methodology		Low methodology		Moderate methodology		Moderate methodology		High methodology	

**Table 3:** **Table 3:** Treatment rendered to the anterior primary intruded teeth

																		*Treatment rendered*					
*SI. no.*		*Author*		*Geographic* * location*		*Total number of* * intruded teeth*		*Lost to* * follow-up*		*Unavailable* *data/not* *analyzed*		*Total number of* * intruded teeth* * assessed*		*Average age* * at trauma*		*Labial bone* * fracture*		*Immediate* * extraction*		*Allowed for* *spontaneous* *re-eruption*		*Repositioned*	
1		Ravn^3^		Copenhagen		88 Complete 80 Partial 8						88		3 years		No data		0		88			
2		Soporowski etal^11^		–		47				17		Spontaneous 21 Repositioned 3		3 years		No data		6		36		*5*	
3		Borum and Andreasen ^14^		Copenhagen		91		18				85		1-5 years		No data		6		85		***―***	
4		Carvalho etal^16^		Rio De Janeio		221 Complete 93 Partial 128		34				187		1-4 years		No data				187			
5		Holan and Ram^17^		Jerusalem		172 Complete 83 Partial 83 Unknown 6						123		2-3 years		Yes 163 No 49		23		149			
6		Gondim and Moreira Neto^18^		Brazil		22		22				22		3 years		No data		–		22		–	
7		Spinas et al^19^		Italy		130		No loss				110		No data		No data		20		110		–	
8		Colak etal^20^		Belgrade		102				7		95		1-6 years		No data		–		95		–	
9		Altun et al^21^		Turkey		169		31				102		2-4 years		No data		36		102		–	
10		Hirata et al^25^		Hiroshima		21						Spontaneous 14		1-4 years		No data				14		7	
						Complete 8						Repositioned 7											
						Partial 13																	

Association of labial bone fracture and spontaneous re-eruption:

Intrusion of primary teeth in some instances may be associated with alveolar fractures. Two studies evaluated the sequelae and found no significant difference.^14,17^

Association of oral habit and spontaneous re-eruption of intruded tooth:

Of three studies, two authors evaluated the presence of oral habits and re-eruption of intruded anterior primary teeth. In both the studies,^17,25^ no significant association was seen between the existence of oral habits and re-eruption.

Association of antibiotics and prognosis of intruded tooth:

Two authors^17,25^ had not found significant improvement in prognosis of intruded primary tooth following systemic antibiotics.

**Table 4:** **Table 4:** Level of re-eruption of intruded anterior primary teeth

										*Level of re-eruption*							
*Sl. no.*		*Author*		*Total number* * of teeth with* * follow-up*		*Duration of* * follow-up*		*Degree of* * intrusion*		*Partial* * re-eruption*		*Complete* * re-eruption*		*No* *re-eruption*		*Teeth remained* * till normal* * exfoliation*	
1		Ravn^3^		88		Avg 3 years				–		90.09% (80)		5% (4)		90.09% (80)	
2		Soporowski et al^11^		Spontaneous 21 Repositioned 3		Avg 4.3 years										No data	
3		Borum and Andreasen^14^		85		Avg 7 years				22.38% (15)		71.64% (48)		5.97% (4)		No data	
4		Carvalho et al^16^		187		Avg 8 years		Complete 120 Partial 67									
5		Holan and Ram^17^		123		Avg 4.5 years		Complete 58 Partial 64 Unknown 1		10% (12) 12% (7) 8% (5)		88% (108) 84% (49) 92% (59)		2% (2) 4% (2) 0		58% (100)	
6		Gondim and Moreira Neto^18^		22		Avg 8 months				47% (10)		42.5% (9)		10.5% (2)		57% (13)	
7		Spinas et al^19^		130		No data				63.6% (70)		13.6% (15)		22.8% (25)		No data	
8		Colak et al^20^		95		Not mentioned						89.48% (85)		10.52% (10)		76.47% (78)	
9		Altun et al^21^		102		Avg 2.7 years				78.4% (80)		14.7% (15)		6.9% (7)		–	
10.		Hirata et al^25^		Spontaneous 14		Avg 7 years		Complete 8		7.14% (1)		92.86% (13)		–		100% (14)	
				Repositioned 7				Partial 13		–		–		–		85.7% (6)	

### Repositioning of Intruded Anterior Primary Teeth

Only two^17,25^ of nine studies considered repositioning of intruded teeth as a treatment option. Hirata et al^25^ mentioned that fixation was done following repositioning of intruded primary teeth. However, the technique of repositioning was not clearly mentioned in both the studies.

### Sequelae of Intruded Anterior Primary Teeth

All the included studies in the review (Table 5) had explored the development of late complications of the re-erupted or repositioned intruded primary teeth. However, there was an overlap of complications, such as pulpal necrosis, pulp canal obliteration, periodon-tal breakdown, ankylosis, coronal discoloration, and resorption developed in teeth. ^3,11,16-18,20,21,25^ Hence, the actual number of teeth that had developed complications cannot be assessed. In four studies, it was seen that around 57% of re-erupted anterior primary intruded teeth survived without complications.^11,18,20,21^ Borum and Andreasen^14^ reported a lower percentage of teeth without complications (11.8%).

Age at time of injury and sequelae developed:

Five of nine studies had shown age of injury as an influencing factor in the development of complica-tions.^11,14,17,20,21^ Pulp canal necrosis was seen in an intruded teeth when the apex was completely closed at time of injury.^11,14,20^ Though Altun et al^21^ had observed pulpal necrosis in children from 1 year of age up to 4 years, maximum pulpal necrosis was seen between 3 to 4 years of age.^21^ Higher occurrence of pulp canal obliteration was seen when teeth had an open apex, i.e., in children below 2 and above 5 years.^14,17^ Incidence of external and internal resorption was seen in children younger than 2 years of age at time of injury.^21^ Carvalho et al^16^ found no significant correlation between age of intrusion and frequency of subsequent complications in the injured tooth.

Degree of intrusion and sequelae of intruded anterior primary teeth:

In five of the nine observational studies, degree of intrusion was considered a factor for the sequelae of intruded anterior primary teeth.^1,16,17,20,25^ It was seen across the studies though partially intruded teeth did not show significant difference in re-eruption, they exhibited relatively lesser degree of complications than completely intruded teeth. Almost 80% of re-erupted teeth showed some pathological complications. Complications, such as discoloration, pulpal necrosis, and premature tooth loss were relatively more in moderately and severely intruded teeth than in mildly intruded teeth.^16,20,25^ Though periodontal breakdown has been reported in few cases across the studies (58 out of 738 teeth), completely intruded teeth have more breakdown than partially intruded teeth.^18^

**Table 5:** **Table 5:** Sequelae of anterior intruded primary teeth

								*Pulpal complications*				*Periodontium complications*				*Coronal discoloration*				*Resorption*			
*Sl. no.*		*Authors*		*Total number of* * teeth with follow-up*		*Degree of* * instrusion*		*Necrosis*		*Pulp canal* * obliteration*		*Periodontal* * break down*		*Ankylosis*		*Dark*		*Yellow*		*Internal*		*External*	
1		Ravn^3^		88		Complete 80 Partial 8		–		37.5% (30)		25% (10)		No data		No data		No data		No data		No data	
2		Soporowski		Spontaneous 21		No data		33.3% (7)		9.5% (2)		No data		0%		No data		No data		No data		No data	
		et al^11^		Repositioned 3				0%		33.3% (1)		No data		33.3% (1)									
3		Borum and Andreasen^14^		85		No data		37.6% (32)		41.1% (35)		No data		2.4% (2)		48.2% (41)				No data		1.2% (1)	
4		Carvalho et al^16^		187		Complete 120 Partial 67		78.9% (101) 23.6% (24)		0.87% (1) 0% (0)		No data		No data		3.1% (4) 17.4% (16)				2.3% (3)		No data	
5		Holan and Ram^17^		123		Complete 58 Partial 64 Unknown 1		No data		64% (33) 40% (24)		37% (15) 63% (25)		No data		17.36% (19+[2-pink])		45.45% (55)		5.79% (7)		No data	
6		Gondim and Moreira Neto^18^		22		No data		23% (5)		0% (0)		Bone loss 19% (4) Mobility 19% (4)		No data		No data		No data				33% (7)	
7		Spians etal^19^		130		No data		No data		No data		No data		No data		No data		No data		No data			
8		Colak etal^20^		95		No data		8.82% (9)		0% (0)		No data		No data		9.8% (10)						2.1% (2)	
9		Altun et al^21^		102		No data		31.4% (32)		0.98% (1)		No data		1.96% (2)		No data		No data				5.88% (6)	
10		Hirata et al^25^		Spontaneous 14		Complete 8		0% (0)		36% (5)		0% (0)		No data		7.14% (1)		28.57% (4)		No data		57.14% (8)	
				Repositioned 7		Partial 13		43% (3)		0% (0)		0% (0)				57.14% (4)		(0)				71.0% (5)	

### Extraction of Intruded Anterior Primary Teeth

Extraction of intruded primary teeth was done either immediately or following development of complica-tions.^11,14,17,19,21^ Teeth considered for immediate extraction were those that were pushed against permanent tooth bud,^17^ excessive mobility,^19^ severe caries lesion, or associated with bone fractures.^14,20,21^ Only 65 of 341 spontaneously re-erupted teeth in the four studies that have been extracted due to development of late complications like periodontal breakdown, pathological root resorption, and caries.^3,17,20,22^ Hirata et al^25^ had reported extraction of repositioned tooth due to development of abscess.

## DISCUSSION

The objective of the present review was to analyze systematically the various treatment modalities and sequelae of re-erupted intruded anterior primary teeth. It will be extremely valuable to the clinicians in the management of intruded primary tooth, and it opens arenas for researchers to conduct further studies. The need to save young primary teeth for cosmetic and space maintenance purpose often outweighs rendering complex treatment and possible posttraumatic sequelae.^19,29^

### Determining the Position of Intruded Primary Incisor

The decision on to extract or to retain the intruded primary tooth depends on the relative position of root of primary tooth and the permanent tooth bud. Due to labial curvature of root apex, there is a high incidence of displacement of root in a labial direction. The incidence is more in 24 to 35 months of age whereas it is reversed to a lower percentage to younger and older children. In contrast, only one-fifth of the roots are displaced palatally.^17^ However, in such instances severe damage occurs to the succedaneous tooth and extraction of primary incisor is advocated. Clinical findings, such as swelling of upper lip, subcutaneous hematoma adjacent to nostrils and in maxillary anterior vestibule, and projected labial bone plate confirmed by palpation establish the fact that the roots of intruded primary incisor are displaced labially.

Radiographs are an important adjunct to the clinical examination to determine the position of the intruded tooth. In a periapical radiograph, if the tooth appears foreshortened compared to noninjured antemere, then one can assume the labial displacement of root with minimal risk to permanent tooth bud and *vice versa.^30^*

In children younger than 20 months, extraoral radiographs can also be the only means to determine the alignment of the root. Investigators are of opinion that it is possible to determine the actual position of intruded primary incisor in an extraoral radiograph only when the root of intruded primary incisor has been displaced labially, accompanied by fracture of labial bone plate.^31^ However, such cases can be diagnosed with clinical signs and periapical radiographs alone. Therefore, the application of extraoral radiographs in diagnosing the position of intruded tooth is limited to certain clinical situations.

Superimposition of structures in primary dentition and two-dimensional view on conventional radiographs lead to a doubtful diagnosis of intruded primary tooth. Cone beam computed tomography (CBCT) is a useful adjunct to determine the exact position of intruded primary tooth and underlying permanent tooth bud.^32^

### Management of Intruded Anterior Primary Teeth

Spontaneous Re-eruption

Watchful waiting therapy has been a common therapeutic approach across the included studies of the review where 42.5 to 92.86% re-erupted completely.^3,11,14,16-21,25^ Majority of intruded primary anterior teeth re-erupts within 1 to 6 months without any pathological conse-quences,^18^ and less than half-teeth with diagnosed complications were indicated for extraction. Partially and completely intruded teeth re-erupt approximately by 4 to 6 months since injury.^20^

Variation in Re-eruption

Less than one-fifth of intruded primary teeth do not re-erupt completely.^14,19,21^ This is due to higher resiliency of alveolar bone surrounding the primary teeth, which protects the periodontal ligament against local damage.^17,21^ Though the reasons remain obscure, fracture of alveolar bone hinders re-eruption as it could lead to scraping of root surface by bone fragment, or tooth gets locked in bone fracture increasing the risk of ankylosis.^14^ Lack of re-eruption can also occur due to partial replacement of dentin pulp complex by functional periodontal liga-ment.^33^ Presence of oral habit has no major influence on re-eruption of intruded primary teeth.^17,25^ Ectopic re-eruption is more often seen in children injured between 24 and 36 months of age, and no significant relation exists between ectopic re-eruption and labial bone fracture.^17^ However, this should not defer the clinician from retaining the primary tooth as in such situations the tooth would only be present in infraocclusion. Later during eruption of permanent incisor a path can be paved by careful extraction of primary tooth.^19^ Hence, allowing an intruded primary tooth for spontaneous re-eruption can be a recommended treatment modality.

### Surgical Repositioning

As spontaneous re-erpution occurs over a period of time and there occurs existence of suspicion, as an alternative treatment modality, surgical repositioning has been suggested in the literature.^1^ Two case reports^34,35^ have shown that immediate surgical repositioning of intruded teeth have shown good prognosis for over an year without development of complications. Among the relevant articles in the present review, two papers have evaluated both surgical repositioning and spontaneous re-eruption. Though with a few case series we cannot assess the benefits of surgical repositioning, it can be done in a situation when there is no impact of primary root on permanent tooth bud, when there is moderateto-severe intrusion, when the child is cooperative, and when both child and parent can be motivated to maintain oral hygiene.^35^

### Sequelae of re-erupted or repositioned Intruded Primary Anterior Teeth

Pulpal Necrosis

Pulpal necrosis is the most common consequence of intrusive injuries in all type of intrusion.^3,11,14,16,18-21,25^ Age of children at the time of injury seemed to be a significant factor in the development of pulp necrosis.^14,21^ Children with severely intruded teeth and those injured in 2 to 3 years old exhibited more pulpal necrosis which is essentially due to mature root apex.^20^ Children lesser than 2 years and greater than 5 years old showed a sequelae-free survival post injury due to the presence of open apex with high vascular supply and shorter pulp.^11^ Soporowski et al^11^ had observed that in a repositioned intruded tooth the occurrence of pulpal necrosis is lesser than spontaneously re-erupted teeth. This is attributed to the fact that repositioning an intruded primary tooth moves the tooth outward, relieving apical compression, lessening the likelihood of ischemia, and allowing for possible reanasto-mosis. On the contrary, Hirata et al^25^ had observed pulpal necrosis in 43% of repositioned intruded tooth unlike in spontaneously re-erupted teeth. It is attributed to either thumb-sucking or pacifier-sucking habit resulting in unnecessary movement of repositioned tooth.

### Pulp Canal Obliteration

Pulp canal obliteration is seen in a maximum of 40% of teeth,^11,14,21,25^ and it is seen more in children below 2 years and above 3 years^17^ unlike pulpal necrosis which occurs between 2 and 3 years.^11,20^ At these ages, the apex may be open, allowing revascularization of the pulp and apposition of calcified deposits. Presence of pulp canal obliteration does not affect the physiologic resorption. Hence, usually does not call for intervention.^14^ But in cases of development of pathological signs endodontic treatment or extraction might be required.

### Coronal Discoloration

Coronal discoloration of a re-erupted primary tooth is yet another common sequelae which causes controversy in treatment plan.^16,17,20^ Discoloration of a re-erupted intruded primary tooth alone is not a reliable indicator of health of pulp. Borum and Andreasen argued that pulpal necrosis cannot be diagnosed solely by discoloration since they observed that 25% of permanently discolored teeth remained without pathological symptoms, and 25% of teeth without color changes showed pulpal necrosis during the follow-up period. Hence endodontic treatment may be unnecessary as there is less than 50% of chance that discolored teeth will become infected and require either puplectomy or extraction. Furthermore, there is no damage to permanent successors as long as pathoses are restricted to pulpal cavity.^36^

### Periodontal Complications

Periodontal Complications During intrusion of teeth, rupture of attachment is unavoidable, leading to easy accessibility of oral bacteria to injured tissues. However, only few intruded teeth have been reported with peri-odontal complications.^3,18^ It can be attributed to higher resilience of alveolar bone surrounding the primary teeth, which protects them against damage to periodon-tal ligament.^14^ Antibiotics at initial visit can reduce the number of extraction of intruded teeth, but no definite guidelines exist for the administration of antibitotics.^37^ On the contrary, maintenance of oral hygiene and cleansing of gingival crevice surrounding the affected teeth with chlorhexidine preparation can cause reduction of oral bacteria.^3,17^

### Resorption

There is no discrete data available from the reviewed studies regarding different types of resorption. Holan^36^ had suggested in cases of internal resorption endodontic treatment can save the tooth. Judicious use of antibiotics, such as penicillin or erythromycin also influences the health of periodontium of intruded tooth to a great extent.^37^ Therefore, it is understood that there is a need for constant monitoring at regular intervals so as to intervene as early as possible to prevent loss of tooth.^19^

### Extraction

Immediate extraction of intruded tooth is indicated when there is a palatal displacement of the primary tooth, leading to damage of the succedaneous tooth bud. Ravn^23^ had observed that 52% of the intruded teeth that had been left for spontaneous re-eruption showed developmental disorders, whereas 72% of teeth presented with structural disorders on permanent tooth when treatment was extraction. Torriani et al^38^ concluded from their study on dogs that the morphological changes in a permanent tooth is an immediate effect to trauma during intrusion than as a sequelae to trauma. However, Thylstrup and Andreasen^39^ had observed no histologic or morphologic differences in the primary teeth that were immediately extracted or left to passively re-erupt. The metaplastic changes in reduced enamel epithelium of permanent tooth bud following injury by an intruded primary incisor are less severe when the tooth is extracted immediately than when the tooth is left in place.^40^ Though animal studies support the view of retaining an intruded primary tooth even if it interferes with permanent tooth bud, human clinical trials are essential to arrive at a conclusive treatment plan. This emphasizes the need for careful manipulation of tissues during extraction. A re-erupted or repositioned intruded tooth calls for extraction only when there are pathological changes that may cause alteration in permanent tooth bud.^19^

In order to examine the effectiveness of management of intruded primary teeth, an ideal situation would be the analysis of randomized clinical trials (RCTs) where groups of patients have been allocated for extraction, observation, and surgical repositioning. In the interest of the patient, it is often not an ethical practice to randomly assign treatment intervention. In situations where RCTs are not feasible, observational studies are the only source of valuable information to literature. Hence, only cross-sectional studies including both retrospective and prospective studies have been included in the systematic review. Literature in languages other than English and those in other databases were not included for the review, which could have resulted in omission of studies relevant to the review.

The limitation of these observational studies is that the children examined and treated by various clinicians may subject to variation in diagnosis and treatment, rendering to intruded anterior primary tooth, and not based on a standardized established protocol. Inclusion of retrospective studies in this systematic review is a limitation as standardized data collection forms were not used across the studies considered for review. Insufficient sample size in the case series could have also influenced the interpretation of the outcome of the study. This could introduce a potential bias among the included studies. In addition, the time of diagnosis of different complication is dependent on the recall interval followed by different authors. Furthermore, few complications could have occurred between last follow-up and exfoliation of tooth. A further limitation is that only maxillary intruded primary teeth have been evaluated in all studies, thereby forcing to make an influence on treatment for other teeth. Various classifications of intrusion have been followed in different studies.^16,20,21^ These could have led the authors of the systematic review interpret the degree of intrusion and management of intruded primary tooth in a nonconclusive manner.

### Implication for Research

A trial with a large number of participants will facilitate in arriving at an accurate decision of the management of intruded primary tooth. As the incidence of intrusion is low, an intrinsic difficulty in conducting a research lies in the sample size.

## CONCLUSION

The successful treatment is influenced by the age of the child, severity and direction of intrusion, presence or absence of caries, fracture of alveolar bone, and time lapse between trauma and dental care. Based on the evaluated studies, it can be concluded that all intruded anterior primary teeth can be allowed for spontaneous re-eruption in absence of evidence of injury to permanent tooth bud. Surgical repositioning is an alternate treatment option when the root is buccally displaced and both parent and child are motivated in maintaining a good oral hygiene. In addition, endodontic treatment can be performed when pulpal or periodon-tal complications develop leading to symptoms like pain, abscess, and pus drainage. Immediate extraction of intruded primary tooth as a treatment option, only when there is permanent tooth injury, needs further research.
